# Accuracy, Utilization, and Effectiveness Comparisons of Different Continuous Glucose Monitoring Systems

**DOI:** 10.1089/dia.2018.0374

**Published:** 2019-03-01

**Authors:** John B. Welsh, Peggy Gao, Mark Derdzinski, Sarah Puhr, Terri Kang Johnson, Tomas C. Walker, Claudia Graham

**Affiliations:** Dexcom, Inc., San Diego, California.

**Keywords:** Continuous glucose monitoring, Dexcom G5, Dexcom G6, Propensity scoring, Real-world evidence.

## Abstract

***Background:*** Accuracy and feature sets of continuous glucose monitoring (CGM) systems may influence device utilization and outcomes. We compared clinical trial accuracy and real-world utilization and effectiveness of two different CGM systems.

***Materials and Methods:*** Separately conducted accuracy studies of a fifth-generation and a sixth-generation CGM system involved 50 and 159 adults, respectively. For between-system performance comparisons, propensity score methods were utilized to balance cohort characteristics. Real-world outcomes were assessed in 10,000 anonymized patients who had switched from the fifth-generation to the sixth-generation system and had used connected mobile devices to upload data from both systems, allowing pairwise comparisons of device utilization and glucose concentration distributions.

***Results:*** Propensity score-adjusted mean absolute relative differences for the fifth- and sixth-generation systems were 9.0% and 9.9%, and the percentages of values within ±20%/20 mg/dL were 93.1% and 92.5%, respectively. The sixth-generation system, but not the fifth-generation system, met accuracy criteria for interoperable CGM systems. Both systems had high real-world utilization rates (93.8% and 95.3% in the fifth- and sixth-generation systems, respectively). Use of the sixth-generation system was associated with fewer glucose values <55 mg/dL (<3.1 mmol/L) (0.7% vs. 1.1%, *P* < 0.001) and more values 70–180 mg/dL (3.9–10.0 mmol/L) (57.3% vs. 56.0%, *P* < 0.001) than the fifth-generation system.

***Conclusions:*** CGM performance outcomes can be compared through the propensity score analysis of clinical trial data and pairwise comparisons of real-world data. The systems compared here had nearly equivalent accuracy and utilization rates. Longer term biochemical and psychosocial benefits observed with the fifth-generation system are also expected with the sixth-generation system.

## Introduction

Worldwide adoption, performance, and usability characteristics of continuous glucose monitoring (CGM) systems continue to improve, and the association between real-time CGM usage and improved outcomes continues to grow. Particular examples of outcomes associated with the G4 PLATINUM and G5 Mobile CGM systems (Dexcom, Inc., San Diego, CA) include A1C reductions in the DIAMOND clinical trial for patients with either type 1 diabetes (T1D)^1^ or type 2 diabetes (T2D),^2^ and reductions in hypoglycemia in the HypoDE clinical trial^3^ for patients with T1D at high risk for severe hypoglycemia. Compared with self-monitored blood glucose (SMBG) therapy, these CGM systems were also associated with improvements in hypoglycemia confidence,^4,5^ diabetes distress,^5^ and glycemic variability.^4^ Accordingly, personal real-time CGM systems are now the standard of care for many individuals with insulin-treated diabetes, and the emerging standard of care for countries outside the United States.^6^

Many CGM system features contribute to the decision to begin CGM or switch from one system to another, including perceived utility and accuracy, ease of use, cost and insurance coverage, and/or reimbursement in certain countries outside the United States.^7^ A sixth-generation “G6” system (Dexcom) was recently introduced that has several differences aimed at improved usability and durability. Compared with the G4 and G5 systems, the G6 system features a simplified and less-painful insertion process,^8^ no required fingerstick calibrations, a thinner transmitter, and a longer duration of use (10 days as opposed to 7). The G6 system also has an alert (enabled by default) that is activated when a glucose value ≤55 mg/dL (<3.1 mmol/L) is predicted within the next 20 min.

Direct accuracy comparisons between different CGM systems are uncommon and typically require patients to wear two^9–13^ or three^14–16^ sensors simultaneously and submit to frequent blood sampling. Because most clinical trials evaluating outcomes associated with CGM use blinded CGM or SMBG regimens as the control group, direct comparisons of outcomes associated with different CGM systems are even less common.

Data from real-world patients before and after transitioning from one CGM system to another therefore represent an unusual opportunity to directly compare short-term glycemic outcomes from systems used in series. Indirect comparisons of system accuracy are possible through retrospective use of propensity scores to adjust point accuracy metrics established in separate clinical trials. To demonstrate that the favorable biochemical and psychosocial outcomes associated with G4 and G5 CGM systems in clinical trials can be expected with the use of the G6 system, we sought to compare their accuracy, utilization rates, and glycemic outcomes.

## Materials and Methods

Accuracy of the G4 system with an advanced signal processing algorithm (“Software 505”) was studied in a clinical trial of adults by comparing CGM data with simultaneously collected “YSI” blood glucose values obtained with the YSI 2300 STAT Plus™ reference instrument (YSI Inc, Yellow Springs, OH).^17^ Because the sensor and algorithm in that study are used in the G5 system, the G5 system has identical accuracy performance characteristics,^18^ and the study is referred to as the “G4/G5 study.” Accuracy of the G6 system was established in separate clinical trials involving the sensor inserted with a manual applicator^19^ or an automated applicator,^20^ both of which used YSI blood glucose values as the comparator.

In the G4/G5 study, subjects were instructed to calibrate their systems twice daily per the labeling recommendation. In both G6 studies, systems were calibrated with SMBG values once daily while in use. However, G6 accuracy metrics were established by subsequent reprocessing of raw sensor data using assigned sensor codes and a factory-calibration algorithm without regard to the SMBG calibration values.

For direct between-system accuracy comparisons, a propensity score method was utilized to balance baseline and demographic characteristics between the two study populations. The propensity model included all available baseline and demographic characteristics shared in the G4/G5 and G6 studies. To achieve optimal balance between two studies, in terms of the propensity scores, interaction terms between correlated factors were also included in the model. Specifically, inverse probability of treatment weighting (IPTW) using the propensity scores was utilized to assess and compare the performances of the systems. Point accuracy metrics from the individual studies (not adjusted for propensity scores) were also evaluated with respect to criteria established by the FDA for integrated CGM (iCGM) systems.^21^ These metrics included the mean absolute relative difference (MARD) between CGM and reference values as well as the percentage of CGM values within 15, 20, or 30 mg/dL (0.8, 1.1, or 1.7 mmol/L) of reference values ≤100 mg/dL (≤5.6 mmol/L) or within 15%, 20%, or 30% of reference values >100 mg/dL (the %15/15, %20/20, and %30/30 metrics, respectively).

Real-world analysis was conducted on data from an anonymized convenience sample of 10,000 G5 users who transitioned to the G6 system between May 1, 2018, and August 31, 2018, and used the corresponding mobile apps to upload and review their CGM data, with no stipulations regarding usage rates or alert settings. Utilization and glucose values for 30 days before and after the first date on which a G6 data point was received were analyzed. Glycemic outcomes are summarized as the percentage of glucose values in various ranges and are compared with pairwise t-tests. As in the ambulatory glucose profile,^22^ data sufficiency is given as the percentage of time that a CGM system was active.

## Results

In the G4/G5 study, the population of 51 adult subjects had a mean (standard deviation [SD]) age of 46.7 ± 15.8 years, and 24 (47%) were women. The mean duration of diabetes was 24.8 ± 14.5 years; 44 (86%) had T1D and 7 (14%) had T2D. All used insulin, but 27 (53%) used pumps and 24 (47%) used multiple daily injections. Average body mass index (BMI) was 27.4 ± 4.6 kg/m^2^, and the mean A1C was 7.8% ± 1.1% (62 ± 12.5 mmol/mol). In the G6 studies, the population of 166 adult subjects had a mean (SD) age of 43.3 ± 15.6 years, and 87 (52.4%) were women. The mean duration of diabetes was 24.5 ± 14.3 years; 161 (97.0%) had T1D and 5 (3.0%) had T2D. All used insulin, but 114 (68.7%) used pumps and 52 (31.3%) used multiple daily injections. The average BMI was 27.8 ± 5.5 kg/m^2^, and the mean A1C was 7.8% ± 1.5% (62.1 ± 16 mmol/mol).

Unadjusted and propensity score-adjusted point accuracy metrics are given in Table 1. The G5 system showed slightly better accuracy than the G6 system with respect to %15/15, %20/20, and MARD, but slightly worse accuracy than the G6 system with respect to %30/30. There were no notable changes when the estimates were adjusted with propensity scores, which may imply that the studies were similar in terms of their designs and patient characteristics.

**Table 1.** Propensity Score-Adjusted Point Accuracy Criteria for the G5 and G6 Systems

*Criterion*	*Unadjusted*		*Adjusted*	
	*G5*	*G6*	*G5*	*G6*
%15/15 (%)	86.1	82.1	86.1	82.1
%20/20 (%)	93.2	92.5	93.1	92.5
%30/30 (%)	98.1	98.9	98.2	98.9
MARD (%)	9.0	9.9	9.0	9.9

MARD between CGM and corresponding YSI values. %XX/XX, percentage of values within XX mg/dL or with XX% of corresponding YSI values for YSI values ≤ and >100 mg/dL (5.6 mmol/L), respectively.

CGM, continuous glucose monitoring; MARD, mean absolute relative difference.

Point accuracy of the G5 and G6 systems was also addressed by comparisons with the special controls for iCGM systems provided by the FDA.^21^ Table 2 shows accuracy rates and the lower one-sided 95% confidence bound of those rates for the two systems studied in adults. The G5 system may not have met all the iCGM criteria (the G5 study preceded the special control requirements). The G5 system does meet iCGM rate of change (ROC) criteria, such that ≤1% of CGM measurements indicated an ROC >1 mg/dL/min (0.06 mmol/L/min) in the presence of a YSI ROC < −2 mg/dL/min (0.11 mmol/L/min), and ≤1% of CGM measurements indicated an ROC < −1 mg/dL/min in the presence of a YSI ROC >2 mg/dL/min.

**Table 2.** G5 and G6 Accuracy Performance in Adults with Respect to Criteria for the Lower One-Sided 95% Confidence Bound of Various Agreement Rates Used in the Determination of Integrated Continuous Glucose Monitoring Status

*CGM range*	*Criteria and performance goals for lower one-sided confidence bound*		*G5 (*n* = 50)*	*G6 (*n* = 159)*
	*Criterion*	*Goal*		
<70 mg/dL (<3.9 mmol/L)	% within ±15 mg/dL (±0.8 mmol/L)	>85%	**90.4 (85.6)**	**88.5 (85.4)**
	% within ±40 mg/dL (±2.2 mmol/L)	>98%	**100.0 (100.0)**	**99.3 (98.7)**
70–180 mg/dL (3.9–10.0 mmol/L)	% within ±15%	>70%	**81.6 (77.4)**	**73.9 (71.3)**
	% within ±40%	>99%	99.3 (98.8)	**99.3 (99.1)**
>180 mg/dL (>10.0 mmol/L)	% within ±15%	>80%	85.4 (79.4)	**85.5 (82.8)**
	% within ±40%	>99%	99.3 (98.2)	**99.9 (99.9)**
Any	% within ±20%	>87%	**91.3 (88.0)**	**89.5 (88.2)**

Data in each cell are the point estimate and the lower one-sided confidence bound in parentheses. Cells in bold indicate that the lower bound meets the performance goal.

Real-world data from 10,000 G5 users who switched to the G6 system are summarized in Table 3. The switch was associated with a small but statistically significant increase in utilization rates and a decrease in mean glucose concentrations. Statistically significant reductions favoring G6 (all *P* < 0.001) were seen in hypoglycemia (a 34% reduction in glucose values <55 mg/dL [<3.1 mmol/L] and a 16% reduction in the glucose values <70 mg/dL [<3.9 mmol/L]) and extreme hyperglycemia (a 7% reduction in glucose values >250 mg/dL [>13.9 mmol/L]). There was also a small increase in the percentage of values 70–180 mg/dL (3.9–10.0 mmol/L) in association with the switch to G6.

**Table 3.** Mean (Standard Deviation) Utilization Rates and Glycemic Parameters for Periods of G5 and G6 Use

	*G5*	*G6*	P
*N*	10,000	10,000	
Utilization rate, %	93.8 (12.0)	95.3 (9.5)	<0.001
Glucose concentration, mg/dL	175.4 (37.8)	173.8 (37.9)	<0.005
Glucose concentration, mmol/L	9.7 (2.1)	9.7 (2.1)	
Glucose values <55 mg/dL (<3.1 mmol/L), %	1.1 (2.0)	0.7 (1.5)	<0.001
Glucose values <70 mg/dL (<3.9 mmol/L), %	3.3 (4.1)	2.8 (3.5)	<0.001
Glucose values 70–180 mg/dL (3.9–10.0 mmol/L), %	56.0 (19.4)	57.3 (20.1)	<0.001
Glucose values >180 mg/dL (>10.0 mmol/L), %	40.7 (20.7)	40.0 (21.2)	0.01
Glucose values >250 mg/dL (>13.9 mmol/L), %	16.2 (14.9)	15.1 (15.1)	<0.001

## Discussion

Comparisons of different medical devices in the absence of head-to-head data may be confounded by differences in the patient populations. Propensity score methods seek to mimic the random assignments of the study patients so that the distribution of measured baseline covariates is similar between the two treatment groups. Each propensity score method has its advantages and disadvantages; hence, it is important to plan and prespecify the method to be utilized before the availability of the outcome information to avoid potential bias. The IPTW method utilizes propensity scores in the analysis stage, which involve the outcome information. However, to reduce any potential bias, all covariates available from both studies were utilized, that is, no additional covariates were collected to be included and no covariate was excluded in the propensity score model.

The performance differences in Table 1 may be due in part to differences in study design, such that a higher proportion of CGM-YSI matched pairs in the hypoglycemic range were observed in the G6 studies than in the G4/G5 study. Despite these small differences, both the G5 and G6 systems are approved as the basis for diabetes treatment decisions in the absence of confirmatory blood glucose measurements. Near-equivalent accuracy of the systems was also seen in the unadjusted agreement rates in Table 2 that are included in the FDA's special controls for iCGM systems.^23^ The iCGM designation reflects several other attributes of the G6 system related to data integrity and alerts, which make it appropriate for use with automated insulin delivery systems in the U.S. market; criteria for this indication in other jurisdictions will likely differ. Because the special controls' accuracy rate criteria are based on the lower 95% confidence interval boundary rather than the point estimate itself, a larger G5 study may have provided data that met more of these stringent performance goals.

The large population of users of the G5 CGM system and the recent introduction of the G6 system provided us with the opportunity to compare glycemic outcomes of different systems used in series, without the need to adjust for different population characteristics. In contrast to the G5 system, the G6 system is unbiased by therapeutic levels of acetaminophen (paracetamol),^24^ does not require fingerstick calibrations, requires fewer steps for sensor insertion, and is designed to last for 10 days instead of 7. The observed hypoglycemia reduction with the G6 system may be partially attributable to its predictive low alert since beneficial reductions in hypoglycemia in association with predictive alerts were noted among users of a CGM system from a different manufacturer.^25^

Device utilization rates in this study were from early adopters of the G6 system. This group may be especially inclined to wear and interact with their CGM devices, so utilization rates may be different for patients who are new to CGM or who have been using the G6 system for more than 3 months. However, device utilization rates have risen markedly in recent years among both adults and children and have been attributed to improvements in accuracy, functionality, and comfort.^26,27^ The absence of patients transitioning from G6 to G5 is another limitation of the study. The study is also limited in that it did not collect data regarding concurrent changes in diabetes therapy, diet, or exercise patterns. The extent to which the transition from G5 to G6 accounted for the favorable changes shown in Table 3 is therefore uncertain.

To the best of our knowledge, this is the first study to combine propensity score-adjusted accuracy metrics and real-world glycemic outcomes in comparing different CGM systems. Given the differences in the G5 and G6 systems with respect to blood glucose testing requirements and sensor life span, longer term studies of health care resource utilization may favor the G6 system. Because of the G6 system's improved usability, equivalent accuracy, and association with improvements in short-term glycemia, benefits such as A1C reduction, hypoglycemia avoidance, and improved quality of life seen in clinical trials of the G4 and G5 systems are expected with the use of the G6 system.
